# Supplementary material for: Inequality can double the energy required to secure universal decent living
Source: Nat Commun. 2022 Aug 26;13:5028. doi: 10.1038/s41467-022-32729-8 (PMC9418152; doi:10.1038/s41467-022-32729-8)
Supplement: Supplementary file 1 — Supplementary Information [file 41467_2022_32729_MOESM1_ESM.pdf]

Supplementary Information for:

Inequality can double the energy required to secure universal decent living

Joel Millward-Hopkins[a,*]

[a]*Sustainability Research Institute, School of Earth and Environment, University of Leeds, Leeds LS2 9JT, UK*
*Corresponding author: joeltmh@gmail.com

Table of Contents

1. Modelling *Fair* Inequalities 2
Idealised distributions 2
Example plot: Inequality in the United Kingdom 2
List of countries studied by inequality category 3
2. Implementing inequalities in material consumption 4
Approach for each sector 4
Nutrition 4
Shelter and living 4
Water 4
Mobility 5
Clothing and Information and Communication 6
Consumption of the super-rich 6
3. The High Population Scenario 6
4. The Current Technology Scenario 7
Comparison with the DLE scenario 7
Nutrition 7
Shelter and living conditions 8
Mobility 8
Power 9
Fixed sectors 9
References 10

1. Modelling *Fair* Inequalities

Idealised distributions

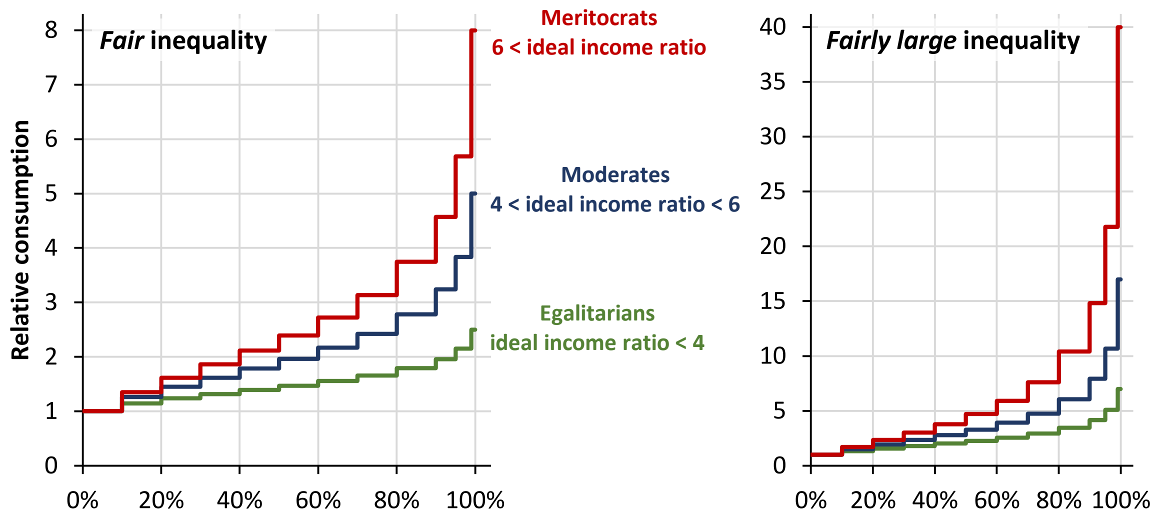

Figure SI1: Idealised distributions of relative consumption for *egalitarian*, *moderate* and *meritocratic* countries in the *fair inequality* scenario (left) and *fairly large* inequality scenario (right). The ranges of *ideal income ratio* in the legend refers to those reported by Kiatpongsan and Norton[1].

Example plot: Inequality in the United Kingdom

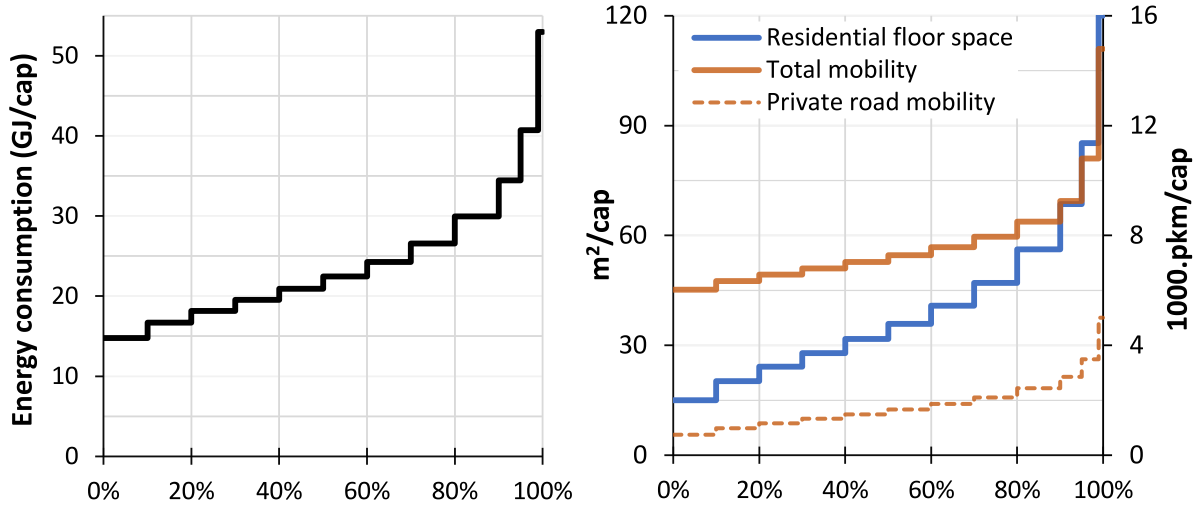

Figure SI2: Final energy footprint inequality across quantiles for the United Kingdom (a *meritocratic* country) in the *fair inequality* scenario (left), and associated inequalities in residential floor-space and mobility (right). Curves for floor space and private road transport mirror the shape of the curve for total energy use on the left, but total mobility is less unequally distributed due to the equality assumed for public transport.

List of countries studied by inequality category

Table SI1: Inequality category of the 39 for which data exist, with the categorisation based upon data from Kiatpongsan and Norton[1].

Egalitarians	Moderates	Meritocrats
1. Philippines	1. New Zealand	1. Australia
2. Argentina	2. Austria	2. China
3. Venezuela	3. Cyprus	3. Japan
4. Belgium	4. Czech Republic	4. Republic of Korea
5. Denmark	5. Estonia	5. Taiwan
6. Latvia	6. Finland	6. United States of America
7. Sweden	7. Hungary	7. Chile
8. Norway	8. Italy	8. France
9. Bulgaria	9. Poland	9. Germany
10. Ukraine	10. Portugal	10. United Kingdom
11. Israel	11. Slovakia	11. South Africa
12. Turkey	12. Slovenia	
	13. Spain	
	14. Switzerland	
	15. Croatia	
	16. Russian Federation	

Table SI2: List of the remaining 81 countries modelled, which are assumed to be *moderates*

Asia:	Africa:	Americas:	Europe:
1. Hong Kong	1. Egypt	1. Canada	1. Greece
2. Mongolia	2. Morocco	2. Mexico	2. Ireland
3. Brunei Darussalam	3. Tunisia	3. Bolivia	3. Lithuania
4. Cambodia	4. Benin	4. Brazil	4. Luxembourg
5. Indonesia	5. Burkina Faso	5. Colombia	5. Malta
6. Lao PDR	6. Cameroon	6. Ecuador	6. Netherlands
7. Malaysia	7. Côte d'Ivoire	7. Paraguay	7. Albania
8. Singapore	8. Ghana	8. Peru	8. Belarus
9. Thailand	9. Guinea	9. Uruguay	9. Romania
10. Viet Nam	10. Nigeria	10. Costa Rica	
11. Bangladesh	11. Senegal	11. Guatemala	
12. India	12. Togo	12. Honduras	
13. Nepal	13. Ethiopia	13. Nicaragua	
14. Pakistan	14. Kenya	14. Panama	
15. Sri Lanka	15. Madagascar	15. El Salvador	
16. Kazakhstan	16. Malawi	16. Dominican Republic	
17. Kyrgyztan	17. Mauritius	17. Jamaica	
18. Armenia	18. Mozambique	18. Puerto Rico	
19. Azerbaijan	19. Rwanda	19. Trinidad and Tobago	
20. Georgia	20. Tanzania		
21. Bahrain	21. Uganda		
22. Iran	22. Zambia		
23. Jordan	23. Zimbabwe		
24. Kuwait	24. Botswana		
25. Oman	25. Namibia		
26. Qatar			
27. Saudi Arabia			
28. United Arab Emirates			

2. Implementing inequalities in material consumption

As described in the main text, the idealised distributions (plotted in Figure SI1) are applied to the dimensions of consumption considered to most closely resemble private luxuries (indicated in Table 1 of the main article). The distributions are taken as dimensionless descriptions of relative consumption and hence applied linearly to the activity-levels of Table 1 (except for food & mobility, as described below). However, it's necessary to check the resulting values are reasonable and apply limits where needed, which represent saturation points for material consumption. The approach is now described for each dimension, and limits are summarised in Table SI3 along with the scenario and country type for which they apply.

Approach for each sector

Nutrition

The default DLE assumptions are that 93% of food energy (kcal) comes from plant-based foods, 3% from meat and the remaining 4% from other animal-based foods, with food-energy consumption between 2,000-2,150 kilocalorie/person/day depending upon countries' age composition. In the inequality scenarios, total food-energy consumption isn't changed, but meat consumption is increased linearly with the relative consumption curves, and assumed to displace plant-based foods. The composition of food consumption is thus changed to be more energy intensive, rather than total consumption increasing.

The change is limited, however, based upon current data. For example, the share of meat (including fish) in food-energy intake in the USA and Australia – where consumption is high – is 13-15%, based on total food supply data[2]. Given these are nationally-averaged values and high-consumers are likely to be well above them, a limit on meat consumption of **20% of food-energy** is applied in the model. As this limit is <7 times the DLE level, it first applies in the *fair inequality* scenario in *meritocratic* countries to the top 1% (who's relative consumption is at 8 times the DLE level; see Figure SI1). Meat consumption is thus the most frequently applied of the limits (see Table SI3).

Shelter and living

The DLE scenario assumes residential floor space of 10m^2/person, with an additional 20m^2/household, thus totalling 15m^2/person. In the inequality scenarios, this is increased linearly with the relative consumption curves, so that in the *fair inequality* scenario in *meritocratic* countries, the top 1% have floor-space of 120m^2/person. This value remains reasonable given new housing in the USA is ~3,000 ft^2 on average for single family homes[3], and more expensive houses will be much larger. Assuming, for example, a 50% larger house size of 4,500 ft^2, this converts to 418 m^2, which for households of 3-4 people is 105-140 m^2/cap.

No limit is thus needed in the *fair inequality* scenario. But in *meritocratic* countries in the *fairly large* inequality scenario, where the relative consumption of the top 1% is 40 times that of the bottom 10%, we apply the same **limit of 500m^2/cap** as used in the *super-rich* scenario (see below).

Water

In the DLE scenario, water consumption is ~50 L/cap/day, with 15 L of this assumed to be heated water for bathing. In the inequality scenarios, this fraction is increased linearly with the relative consumption curves, up to **a limit of 300 L/cap/day**. This is based upon the observation that modern luxury showerheads have a flowrate of 30 L/min, meaning a 10 minute shower would use 300 L of water – 20 times DLE consumption, or as much as 3 average baths. More common in hotels are

luxury showerheads with flowrates of 10-20 L [4], which, assuming a 20 minute shower (or bi-daily 10 minute showers) would total the same 300 L/cap/day.

Mobility

In the DLE scenario, total annual km/cap ranges from 4,900 to 15,000 km/cap, with variation defined as a function of regional population density, and mobility set consistently higher in rural areas[5]. After removing non-motorised and air travel from this total (assumed to be ~1,000 km/cap each), the remaining kilometres are divided between rail, buses and cars (40%, 40% and 20%, respectively). In the inequality scenarios, kilometres by car are increased linearly with the relative consumption curves, and those by train and bus are decreased accordingly. In cases when car travel fully displaces train and bus travel (i.e. when the relative consumption curve exceeds 5, as 20% × 5 = 100%), total mobility is then increased to account for further increases in car travel. In contrast, air travel is increased linearly with no such displacement effect.

The resulting values for both car and air travel appear reasonable. For *meritocratic* countries in the *fair inequality* scenario, annual car travel for the top 1% in the most sparsely populated countries is ~13,000 km/cap to 20,000 km/cap (for urban and rural populations, respectively). The latter is less than the current distances travelled per person by car in the USA[6]. Annual air travel for *meritocratic* countries in the *fair inequality* scenario is equivalent to ~3 short-haul return trips per year; far less than many currently fly.

For *meritocratic* countries in the *fairly large* inequality scenario, linearly increasing car travel would be unrealistic for the top 1%, so we apply the same limits as used in the *super-rich* scenario for car travel (see details below). For air travel, however, a linear increase with no limits remains reasonable, as it leaves the top 1% flying the equivalent of ~2-4 long-haul return trips per year.

Table SI3: The limits (or 'saturation points') defined for consumption categories, and indication of the scenarios for which these limits apply (highlighted red). Some of the limits intended for the *Super-rich* scenario (identified by the symbol 'Δ') also apply in the *Fairly large* inequality scenario for *meritocratic* countries, leaving consumption of the top 1% in these countries at the super-rich level described in the main paper, even outside of the *Super-rich* scenario. However, there are only 11 countries in which this occurs. Note that *E* = *egalitarians*, *Mo* = *Moderates* and *Me* = *Meritocrats*, with the top 1% to bottom 10% ratios in brackets, as reported in the main text.

**Consumption category**		**Limits**	**Scenarios where limits apply**						
			Fair			*Fairly large*			*Super-rich*
			**E (2.5)**	**Mo (5)**	**Me (8)**	**E (7)**	**Mo (17)**	**Me (40)**	
**Nutrition**	Meat consumption	20% of kcal							
**Shelter & living**	Floor-space	500 m^2/cap							Δ
	Hot water	300 L/cap/day							
**Mobility**	Private road travel	40,000 km/yr							Δ
	Air travel	1 flight/week							Δ
**Clothing**	Clothing items	30 kg/cap/yr							
**Info & Comms**	Computer ownership	2.5/cap							

Clothing and Information and Communication

The key DLE assumptions for these sectors are that: each person needs 3 kg of new clothing per year; everyone over 10 years of age owns a smartphone; and there is a laptop in each household (i.e. one per four people). In the inequality scenarios, we increase both the amount of new clothing and laptop ownership linearly with the relative consumption curves, but up to **limits of 10 times the default values**.

These limits are chosen fairly arbitrary compared to the limits defined for other sectors, due to a lack of any more objective way to estimate saturation points. However, they're calibrated so the resulting maximum consumption seems reasonable. For example, 30kg of clothing is roughly one new item per week. Increasing laptop ownership by a factor of ten can be interpreted as increasing the basic laptop ownership of 1/household to 1/person and replacing them 2.5 times more often, or by increasing ownership to 2/person while replacing slightly more often. In any case, these limits are not reached in any country in the *fair inequality* scenario: they are reached only in the *fairly large* inequality scenario – by the top 5% in *moderate* countries and top 20% in *meritocratic* counties – and the top 1% in the *super-rich* scenario.

Consumption of the super-rich

Otto et al.[7] argue that research into environmental impacts has devoted insufficient attention to high net worth individuals – e.g. those with assets totalling over US$1 million, of which there are 36 million globally (about 0.5% of the world's population). To this end, they offer data on mobility and housing activity of a small number of *super-rich* households. The data are wide ranging, showing 1-10 return flights per month, 2,000-4,500 km of driving per month, and residential floor space totalling 200-745m^2 per person when first and second homes are combined[7]. For the *Super-rich* scenario, values roughly within the middle of these ranges are used, specifically, **1 flight/week**, private road transport of **40,000 km/yr**, and household size of **500 m^2**. In the other consumption categories, the same limits as for other two inequality scenarios are applied. This is, of course, a crude and uncertain way of integrating the impacts of super-consumers, but it nonetheless serves to capture the potential impact of such extreme, long-tailed inequality on global ecological impacts.

3. The High Population Scenario

The original decent living energy model used the central population projection of the United Nations population division, which gave a global population of 9.74 billion in 2050. Although this is their central estimate, it is towards the higher end of those in the literature. The projections of Vollset et a. (2020) range from around 8.7 billion to over 10 billion in 2050, with a reference scenario peaking at 9.7 billion but only in 2064[8]. Bradshaw and Brook (2014) present central scenarios with a 2050 population of 9.2-9.3 billion, along with explicitly more extreme scenarios such as a draconian 1-child policy reached by 2045, which results in a 2050 population of 7.6 billion [9]. Abel et al. (2016) suggest that meeting the SDGs would lead to a 2050 population of roughly 8.7-9 billion[10].

The IPCC's SSP population scenarios span this range reasonably well for 2050 – if the extreme scenarios of Bradshaw and Brook (2014) are discounted – with SSP1 and SSP5 at 8.5-8.6 billion, SSP2 and SSP4 at 9.1-9.2 billion, and SSP3 at 10 billion [11]. Consequently, SSP1 is chosen for the *DLE* scenario, and SSP3 for the *high population* scenario.

4. The Current Technology Scenario

Comparison with the DLE scenario

The previous *DLE* model assumed global deployment of highly-energy-efficient, state-of-the-art technologies that are currently available or most likely will be before 2050[5]. Energy intensities were derived via analysis of a broad range of literature, including life cycle assessment, input–output analysis, industrial ecology and state-of-the-art engineering work. The same technological assumptions are used in the current work across the *Decent Living Energy*, *High Population*, *Fair Inequality*, *Super-Rich* and *Fairly Large Inequality* scenarios, and across all technologies with the exceptions of public and private road transport – for these, the energy intensities from the previous *DLE* model are decreased based upon more recent literature, as described below.

An analogous process is then undertaken to develop the *Current Technology* scenario (***CT***), in which ambition is reduced so energy-efficiencies reflect current widely used, but best-practice technologies. This is described below.

Nutrition

For **food production & supply**, I retreat upon the *DLE* assumptions marginally, as the intensities used there were relatively conservative. For production of crops, I assume 4.5 units of food-energy out per industrial-unit in for the *CT* scenario, which is current global best-practice[12] and equates to an energy requirement of **0.22 GJ per 1 GJ of food**; slightly higher than the assumed *DLE* value of 0.2 GJ in/GJ out. For animal-based foods, I assume the same production energy intensities as in the *DLE* model – **3 GJ in/GJ out** and **1.5 GJ in/GJ out** for meat and other animal products, respectively – which mirror UK efficiencies and are thus much higher than in the USA or the global average. Overall supply chain intensities are increased, however, by increasing the energy intensity of processing. In the *DLE* model the energy use of processing was assumed to be 50% of that used in food production itself, after a ~25% reduction based upon an FAO estimate for possible efficiency improvements. For the *current technology* scenario I remove this reduction, thus making processing **~65% of the energy used for food production**.

For **household cooking**, current IEA energy use data can be used[6], which gives PJ for total direct residential end-use by region for various purposes, including cooking. The lowest values are found in the EU and USA (~1.1 GJ/cap/yr), which can be converted to 2 KJ/kcal, assuming 1,500 kcal/cap/day of food is cooked in the home[1]. This is a rough estimate, given uncertainty in the amount of food that is cooked at home. Nonetheless, it seems reasonable – it's ~50% of the global average suggested by the FAO and 2.5 times larger than the *DLE* value (0.8 KJ/kcal) – and hence this **2 KJ/kcal** is used for the CT scenario.

For **household cold storage**, the *DLE* value was based upon the highest practical efficiency improvements possible as suggested by Cullen et al.[13], which imply direct energy consumption of 120 kWh/yr per appliance. Cullen et al. also report that new cold storage appliances in the USA in 2001 had an average energy use of 450 kWh/year. Currently, many high-efficiency cold storage appliances that are A-rated on the EU energy labelling scale have energy usage of around **250 kWh/yr**; I take this value as current best practice for the *CT* scenario.

[1] This 1,500 kcal comes from downscaling a food supply of 3,300 kcal/cap/day, which is given in the FAO food balances, by assuming 20% is uncooked food waste, 33% of food is consumed outside the home, and 20% of remaining consumption is not cooked.

Shelter and living conditions

For **thermal comfort** in homes, the *DLE* model used data from the Global Performance Buildings Network[14]. This offers final energy use for both space heating and cooling across different climate zones based upon *Heating* and *Cooling Degree Days*; for urban and rural buildings; for various building types (residential, hotels, educational, etc.); and for different technology standards. The *DLE* model used the technology standards with the lowest energy use, namely *advanced new buildings*. In contrast, for the *CT* scenario I use the data for the category *moderately advanced new builds & deep retrofits*. Accordingly, while in the *DLE* scenario final energy use ranged from 19 to 55 MJ/m^2 depending upon climate, intensities in the *CT* scenario are 3-4 times higher on average and range from **46-262 MJ/m^2**. I take an identical approach for commercial and public buildings, leading to the ranges reported in the table below.

For the energy embodied in **residential construction**, data in the literature vary substantially. The *DLE* model used values at the low end of that found in three papers reviewing timber-, concrete- and steel-based construction. A value of 30 $MJ/m^2/yr$ was assumed for regions where sufficient timber is available for construction, and 50 $MJ/m^2/yr$ where more energy-intensive materials must instead be used (concrete & steel). Values not much larger than these (i.e. ~50-75 $MJ/m^2/yr$) are suggested even for houses designed for low operational energy use[15]. In the *CT* scenario I increase the *DLE* values 50%, giving **45 and 75 $MJ/m^2/yr$** as the regionally dependent energy intensities of construction. Again, I take an identical approach for commercial and public buildings, increasing the *DLE* values by 50%.

For **illumination**, the *DLE* model assumed highly efficient LEDs with an efficacy of 150 lm/W. For the *CT* scenario, I assume more moderate efficiency LEDs are deployed with efficacy of **100 lm/W**, which is nonetheless much higher than traditional bulbs.

For **water heating**, I use the same heat transfer efficiency of 95% as in the *DLE* model, while assuming a point-of-use system with no distribution losses, but I raise the boiler outlet temperature from 50°c to 65°c to reflect more typical current operation. This raises the energy intensities in the *CT* scenario across the regions – which vary with ambient temperature in the *DLE* model – from 96-220 KJ/L to **162-287 KJ/L**.

For **water supply** – the infrastructure required to deliver water to homes – I use the same energy intensities as in the *DLE* scenario, as these were already based upon widely observed current values.

Mobility

For **private surface transport** (i.e. cars), I set the *CT* scenario direct energy intensity equal to that used in the previous DLE model, which was relatively conservative and in line with widely used current technologies. Specifically, the previous *DLE* direct energy intensity for cars was **0.34 MJ/pkm**, which is close to the value reported by Onat et al.[16] for various current electric vehicles[2] and a little less that reported by Chester et al.[17, 18] for current high-efficiency ICE vehicles (55mpg)[3]. The *DLE* energy intensity is updated based upon data in Cox et al.[19], which offers a long-term (most-likely) estimate of ~15 kWh/100km for future electric vehicles with autonomous control implemented to improve efficiency. This converts to **0.16 MJ/pkm** assuming an occupancy rate of 3.

---

[2] They report 2.4 MJ/km of primary energy per vehicle, which converts to 0.33 MJ/pkm of final energy assuming an occupancy rate of 3.

[3] Their data implies around 50 MJ/pkm assuming an occupancy rate of 3.

For **public road transport** (i.e. buses), I set the *CT* scenario direct energy intensity equal to that used in the previous DLE model, for the same logic as for cars. The previous *DLE* value was **0.18 MJ/pkm**, which matches that achieved in some regions by 2050 in the IEA's 2DS scenario when more efficient technologies are assumed to have become widespread, but remains significantly higher than reported for efficient electric buses today. Lajunen[20] and Lajunen and Liperman[21] suggest 1 kWh/km for current electric buses with 50 person capacity is possible, which converts to **0.072 MJ/pkm**; I take this value for the *CT* scenario.

For **rail transport**, the previous *DLE* value for direct energy is maintained for the current *DLE* scenario (0.057 MJ/pkm), and the *CT* scenario estimate is taken to be three times this at **0.17 MJ/pkm**. This is based upon the IEA 2DS scenario, which projects the energy intensity of rail to remain roughly constant for 2020-2050 at 0.16-0.18 MJ/pkm.

The direct energy intensity of **air transport** is not changed for the *CT* scenario, as technological development in this sector is particularly difficult to estimate. For example, passenger aircraft in the 2000s were only as efficient as those of the 1950s, due to the invention and widespread deployment of jet engines, which replaced the earlier propeller-driven aircrafts[22]. The direct intensities of **freight vehicles** are also not changed for the *CT* scenario, as the IEA 2DS scenario suggests the *DLE* values may be achieved globally by 2050.

For the indirect energy use in vehicle production, the previous *DLE* model applied a 33% reduction in energy intensities for all vehicles based upon a range of manufacturing improvements suggested by Allwood et al.[23] for steel. Effectively, this took steel production as a proxy for the intensity of future vehicle production. For the *CT* scenario I remove this 33% improvement, thus obtaining intensities recorded in **Table SI4** for passenger and freight vehicles.

Power

The DLE model used data from Hertwich et al.[24] to estimate the energy intensity of producing power generation infrastructure. They suggested 0.1-0.25 kWh of energy was required for every 1 kWh of electricity produced, depending upon the renewable technology used. The *DLE* model used **0.15 kWh/kWh** to reflect a mix of generation technologies and I maintain that value here. For the *CT* scenario I increase this to **0.175 kWh/kWh** to be in the middle of the Hertwich et al. range.

Fixed sectors

For three sectors, the *DLE* and *CT* intensities are assumed equal:

For **clothing** and the direct energy use of washing and drying, the *DLE* values are already representative of A-rated washer-dryers: current market data suggests these require ~0.67 kWh/kg of clothes, which matches the 2.4 MJ/kg assumed in the *DLE* scenario. Similarly, for clothes production the *DLE* model already assumed commonly found best-practice values, so these too are retained for the *CT* scenario.

For **communication & information** the values the *DLE* model assumed represent current devices and network infrastructures. These are retained for the *CT* scenario as efficiency improvements in this sector typically serve to support improvements in computing performance rather than reductions in energy use[25]. This trend is, of course, an example of a direct rebound effect, such as when improvements to car's fuel efficiency result in more driving. However, for driving, the distance covered per person is capped in the model at levels assumed to be sufficient for decent living. In contrast, it is not at all clear how one would estimate the speed of communication and information

technologies required for decent living. Only a partial answer can be suggested, namely: a similar speed to those one is socially involved with.

For **waste management**, the *DLE* model took a crude approach of assuming the energy required to be equal to that used for water supply. A more detailed approach was considered infeasible given the difficulty in estimating waste generation in a world with such fundamentally different consumption patterns. I thus use the same values for both the *DLE* and *CT* scenarios.

Table SI4: This includes all parameters that are modified for the *CT* scenario. Parameter that are unchanged are not listed. Note, direct here is energy used directly by households. (Note: the intensity for production of planes includes production of fuels, which is fixed in the *DLE* and *CT* scenarios, hence why the latter value is less than 50% larger than the former.)

Sector and activity		Energy intensity		Units	Energy scope
		DLE	Current technology		
Nutrition	Crop production	0.2	0.22	*GJ in/GJ out*	*Indirect*
	Food processing	50	65	*% of production*	*Indirect*
	Cooking appliances	0.8	2	*KJ/kcal*	*Direct*
	Cold Storage	120	250	*kWh/yr*	*Direct*
Shelter & living conditions	Thermal comfort	19-55	46-262	*MJ/m²/yr*	*Direct*
	Construction	30 & 50	45 & 75	*MJ/m²/yr*	*Indirect*
	Illumination	150	100	*lm/W*	*Direct*
	Water heating	96-220	162-28	*KJ/L*	*Direct*
Public services	Hospitals thermal comfort	68-93	181-361	*MJ/m²/yr*	*Direct*
	Schools thermal comfort	51-66	126-232	*MJ/m²/yr*	*Direct*
	Hospitals construction	273-455	410-682	*MJ/m²/yr*	*Indirect*
	Schools construction	90-150	135-225	*MJ/m²/yr*	*Indirect*
Mobility	Cars propulsion	0.16	0.34	*MJ/pkm*	*Direct*
	Buses propulsion	0.072	0.18	*MJ/pkm*	*Direct*
	Rail propulsion	0.057	0.17	*MJ/pkm*	*Direct*
	Car production	0.1	0.14	*MJ/pkm*	*Indirect*
	Buses production	0.04	0.06	*MJ/pkm*	*Indirect*
	Rail vehicle production	0.007	0.010	*MJ/pkm*	*Indirect*
	Plane production	0.18	0.22	*MJ/pkm*	*Indirect*
Commercial activity	Road freight vehicle production	0.11	0.16	*MJ/tkm*	*Indirect*
	Rail freight vehicle production	0.009	0.014	*MJ/tkm*	*Indirect*
	Building thermal comfort	45-58	159-244	*MJ/m²/yr*	*Direct*
Power	Infrastructure	0.15	0.175	*kWh/kWh*	*Indirect*

References

1. Kiatpongsan, S. and M.I. Norton, *How Much (More) Should CEOs Make? A Universal Desire for More Equal Pay.* Perspectives on Psychological Science, 2014. **9**(6): p. 587-593.
2. FAO, *New Food Balances*, UN, Editor. 2018.
3. Moura, M., S. Smith, and D. Belzer, *120 Years of U.S. Residential Housing Stock and Floor Space.* PLOS ONE, 2015. **10**(8): p. e0134135.
4. Styles, D., H. Schönberger, and J. Galvez Martos, *Best Environmental Management Practice in the Tourism Sector*. 2017, European Commission.

5. Millward-Hopkins, J., et al., *Providing Decent Living with Minimum Energy: A Global Scenario.* Global Environmental Change, 2020. **65**: p. 102168.
6. IEA, *Energy Technology Perspectives 2017: Catalysing Energy Technology Transformations* 2017, OECD/IEA.
7. Otto, I.M., et al., *Shift the focus from the super-poor to the super-rich.* Nature Climate Change, 2019. **9**(2): p. 82-84.
8. Vollset, S.E., et al., *Fertility, mortality, migration, and population scenarios for 195 countries and territories from 2017 to 2100: a forecasting analysis for the Global Burden of Disease Study.* The Lancet, 2020. **396**(10258): p. 1285-1306.
9. Bradshaw, C.J.A. and B.W. Brook, *Human population reduction is not a quick fix for environmental problems.* Proceedings of the National Academy of Sciences, 2014. **111**(46): p. 16610.
10. Abel, G.J., et al., *Meeting the Sustainable Development Goals leads to lower world population growth.* Proceedings of the National Academy of Sciences, 2016. **113**(50): p. 14294-14299.
11. Kc, S. and W. Lutz, *The human core of the shared socioeconomic pathways: Population scenarios by age, sex and level of education for all countries to 2100.* Global Environmental Change, 2017. **42**: p. 181-192.
12. Pellegrini, P. and R.J. Fernández, *Crop intensification, land use, and on-farm energy-use efficiency during the worldwide spread of the green revolution.* Proceedings of the National Academy of Sciences, 2018. **115**(10): p. 2335-2340.
13. Cullen, J.M., J.M. Allwood, and E.H. Borgstein, *Reducing Energy Demand: What Are the Practical Limits?* Environmental Science & Technology, 2011. **45**(4): p. 1711-1718.
14. GBPN, *Tool for Building Energy Performance Scenarios*, G.B.P. Network, Editor. 2012: Centre for Climate Change and Sustainable Energy Policy (3CSEP), Central European University.
15. Sartori, I. and A.G. Hestnes, *Energy use in the life cycle of conventional and low-energy buildings: A review article.* Energy and Buildings, 2007. **39**(3): p. 249-257.
16. Onat, N.C., M. Kucukvar, and O. Tatari, *Conventional, hybrid, plug-in hybrid or electric vehicles? State-based comparative carbon and energy footprint analysis in the United States.* Applied Energy, 2015. **150**: p. 36-49.
17. Chester, M., et al., *Infrastructure and automobile shifts: positioning transit to reduce life-cycle environmental impacts for urban sustainability goals.* Environmental Research Letters, 2013. **8**(1): p. 015041.
18. Chester, M. and A. Horvath, *High-speed rail with emerging automobiles and aircraft can reduce environmental impacts in California's future.* Environmental Research Letters, 2012. **7**(3): p. 034012.
19. Cox, B., et al., *Uncertain Environmental Footprint of Current and Future Battery Electric Vehicles.* Environmental Science & Technology, 2018. **52**(8): p. 4989-4995.
20. Lajunen, A., *Lifecycle costs and charging requirements of electric buses with different charging methods.* Journal of Cleaner Production, 2018. **172**: p. 56-67.
21. Lajunen, A. and T. Lipman, *Lifecycle cost assessment and carbon dioxide emissions of diesel, natural gas, hybrid electric, fuel cell hybrid and electric transit buses.* Energy, 2016. **106**: p. 329-342.
22. Peeters, P., J. Middel, and A. Hoolhorst, *Fuel efficiency of commercial aircraft: An overview of historical and future trends*. 2005, National Aerospace Laboratory, the Netherlands.
23. Allwood, J., et al., *Sustainable materials: with both eyes open*. 2012: Citeseer.
24. Hertwich, E.G., et al., *Integrated life-cycle assessment of electricity-supply scenarios confirms global environmental benefit of low-carbon technologies.* Proceedings of the National Academy of Sciences, 2015. **112**(20): p. 6277-6282.
25. Williams, E., *Environmental effects of information and communications technologies.* Nature, 2011. **479**: p. 354.
